# Sodium butyrate reverses lipopolysaccharide‐induced mitochondrial dysfunction in lymphoblasts

**DOI:** 10.1111/jcmm.17342

**Published:** 2022-05-19

**Authors:** Scott L. Weiss, Donglan Zhang, Sumera Farooqi, Douglas C. Wallace

**Affiliations:** ^1^ Department of Anesthesiology and Critical Care Children's Hospital of Philadelphia University of Pennsylvania Perelman School of Medicine Philadelphia Pennsylvania USA; ^2^ Pediatric Sepsis Program at the Children's Hospital of Philadelphia Philadelphia Pennsylvania USA; ^3^ Center for Mitochondrial and Epigenomic Medicine at the Children's Hospital of Philadelphia Philadelphia Pennsylvania USA; ^4^ Department of Pediatrics Children's Hospital of Philadelphia University of Pennsylvania Perelman School of Medicine Philadelphia Pennsylvania USA

**Keywords:** human immunology, immunometabolism, metabolism, mitochondria, sepsis

## Abstract

Butyrate is a short‐chain fatty acid that is produced by commensal microbes within the intestinal microbiome through fermentation of dietary fibre. Microbial‐derived butyrate has been shown to promote immunologic and metabolic homeostasis, in part through its beneficial effects on mitochondrial function, and thus has been proposed as a possible anti‐inflammatory therapy. We tested the hypothesis that butyrate could mitigate the decrease in mitochondrial respiration in immune cells under septic conditions as a preliminary step towards better understanding the potential for butyrate as a novel therapy in sepsis. Mitochondrial respiration and content (measured as citrate synthase activity) were compared within four Epstein–Barr virus‐transformed lymphoblast (LB) cell lines exposed to either control media or lipopolysaccharide (LPS) 100 ng/ml. Both co‐incubation of LBs with LPS + butyrate and treatment with butyrate after LPS stimulation reversed the decrease in mitochondrial respiration observed in LBs exposed to LPS without butyrate. Neither LPS nor butyrate led to significant changes in citrate synthase activity. The preliminary findings support further investigation of a potential mitochondrial‐based mechanism through which butyrate may help to mitigate the immuno‐inflammatory response in sepsis.

## 1 INTRODUCTION

Sodium butyrate is a short‐chain fatty acid produced through fermentation of dietary fibre by microbiota of the large intestine, most notably of the Firmicutes phylum.^1^ Microbial‐derived butyrate supports mucosal integrity and limits inflammation in the colon,^2^, ^3^ and systemic absorption aids immune and metabolic homeostasis.^4^, ^5^ Such favourable effects may be lost in critical illness due to a reduction in butyrate‐producing microbes from the microbiome. Supplementation of butyrate in animal models of sepsis can inhibit inflammation, maintain intestinal barrier function, augment memory and improve survival,^2^, ^3^, ^4^, ^6^ while more generalized efforts to restore the microbiome through faecal microbiota transplant found that expansion of butyrate‐producing microbes plays a key role in the observed benefits of this therapy.^7^

One mechanism through which butyrate may impact health is its effect on mitochondrial function. In a study of children with sepsis, we found that loss of Firmicutes microbes was associated with lower levels of stool butyrate, which in turn correlated with decreased mitochondrial respiration in peripheral blood mononuclear cells (PBMC).^8^ Previously, we had shown that low PBMC mitochondrial respiration beyond Day 3 of hospitalization was more common in children with prolonged organ dysfunction, sustained inflammation and immune paralysis.^9^ However, whether butyrate can directly improve PBMC mitochondrial function is not known. Therefore, we sought to test the hypothesis that butyrate could mitigate the decrease in mitochondrial respiration in immune cells under septic conditions as a preliminary step towards better understanding the potential for butyrate as a novel therapy in sepsis.

## 2 METHODS

We exposed four Epstein–Barr virus (EBV)‐transformed lymphoblast (LB) cell lines to incubation in either control media (RPMI 1640) alone or addition of lipopolysaccharide (LPS) 100 ng/ml (Sigma‐Aldrich L5886) for 4 h at 37°C in 5% CO_2_.

In the first experiment, LBs were incubated for 4 h with either control media or LPS supplemented with increasing concentrations of sodium butyrate (Sigma B5887) of 0, 0.1, 0.5 and 1.0 mM. Concurrent exposure to butyrate and LPS was performed to test the ability of butyrate to prevent LPS‐induced changes in mitochondrial respiration. Butyrate concentrations between 0.1 and 1 mM simulate the concentrations absorbed into the circulation from the colon.^10^ After the 4‐h incubation, LBs were isolated and re‐suspended in fresh RPMI 1640 to rest for 24 h at 37°C in 5% CO_2_ prior to study measurements.

In the second experiment, LBs were incubated for 4 h with either control media or LPS. After the 4‐h incubation, LBs were re‐suspended in fresh RPMI supplemented with increasing concentrations of sodium butyrate for 24 h at 37°C in 5% CO_2_. Treatment with butyrate after exposure to LPS tested the ability of butyrate to reverse the impact of LPS on mitochondrial respiration.

Basal mitochondrial respiration, respiration supporting mitochondrial ATP synthesis (ATP‐linked respiration), and maximal uncoupled respiration through the electron transport system (ETS_max_) were measured in intact LBs using a high‐resolution oxygraph (Oroboros Instruments) with subtraction of non‐mitochondrial respiration from all parameters, as previously described.^9^ Data are reported as oxygen consumption rate (OCR) in pmol/s/million cells. Mitochondrial content was estimated using citrate synthase (CS) activity measured with spectrophotometry, as previously described.^11^ Each experimental condition was repeated four times (one time in each of four LB cell lines). We used paired *t*‐tests to compare differences in LB cell mitochondrial respiration across conditions to account for the same four LB cell lines in each group.

## 3 RESULTS

**FIGURE 1:**
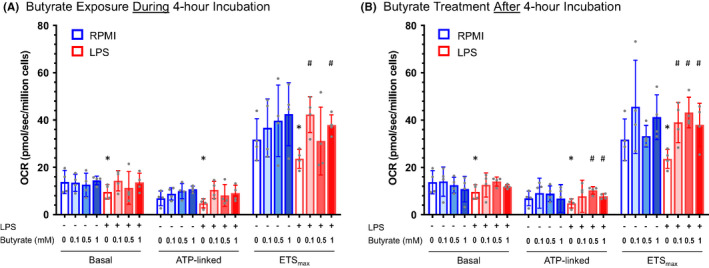
Mitochondrial respiration in lymphoblasts exposed to LPS and butyrate. (A) EBV‐transformed lymphoblasts (*n* = 4 different cell lines per bar) were incubated with either control media (blue) or lipopolysaccharide (LPS, red) without butyrate or with 0.1, 0.5, or 1 mM sodium butyrate for 4 h. Mitochondrial respiration was measured as the oxygen consumption rate in pmol/s/million cells 24 h after the initial incubation. In the absence of butyrate, basal, ATP‐linked, and maximal respiratory capacity (ETS_max_) were significantly lower with LPS incubation (*p < 0.05 compared to control media). Respiration increased in LPS‐exposed cells with addition of butyrate (^#^ *p* < 0.05 compared to LPS without butyrate). (B) EBV‐transformed lymphoblasts (*n* = 4 different cell lines per bar) were incubated with either control media (blue) or lipopolysaccharide (LPS, red) for 4 h. Cells were then transferred to control media alone or media supplemented with 0.1, 0.5, or 1 mM sodium butyrate for 24 h, followed by measurement of mitochondrial respiration. In the absence of butyrate, basal, ATP‐linked, and maximal respiratory capacity (ETS_max_) were significantly lower with LPS incubation (*p < 0.05 compared to control media). Respiration increased in LPS‐exposed cells that were subsequently treated with butyrate (^#^ *p* < 0.05 compared to LPS without butyrate)

**FIGURE 2:**
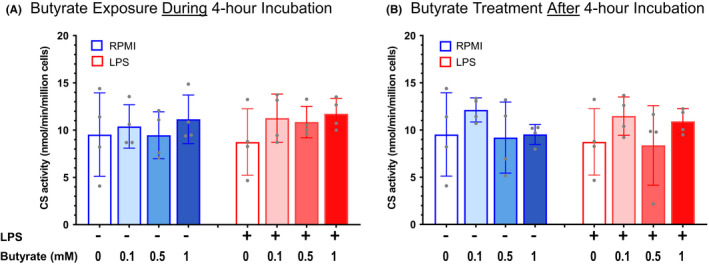
Citrate synthase activity in lymphoblasts exposed to LPS and butyrate. (A) EBV‐transformed lymphoblasts (*n* = 4 different cell lines per bar) were incubated with either control media (blue) or lipopolysaccharide (LPS, red) without butyrate or with 0.1, 0.5 or 1 mM sodium butyrate for 4 h. Citrate synthase (CS) activity was measured as nmol/min/million cells 24 h after the initial incubation. (B) EBV‐transformed lymphoblasts (*n* = 4 different cell lines per bar) were incubated with either control media (blue) or lipopolysaccharide (LPS, red) for 4 h. Cells were then transferred to control media alone or media supplemented with 0.1, 0.5 or 1 mM sodium butyrate for 24 h, followed by measurement of CS activity. CS activity did not change significantly with exposure to either LPS or butyrate

After the initial 4‐h incubation, LPS significantly reduced basal, ATP‐linked and ETS_max_ respiration in the absence of butyrate (Figure 1A). ETS_max_ increased significantly in LPS‐exposed cells in the presence of 0.1 and 1 mM butyrate compared to incubation without butyrate. A similar increase was seen in basal and ATP‐linked respiration in the LPS group with exposure to butyrate, although these increases were not statistically significant. After addition of each concentration of butyrate, mitochondrial respiration was not different between LBs incubated in control media or LPS. CS activity, as a measure of mitochondrial content, was not different between LBs incubated with control media or LPS and did not change with addition of butyrate (Figure 2A).

For LBs treated with butyrate after the initial incubation with either control media or LPS, ATP‐linked respiration increased significantly with 0.5 and 1 mM butyrate and ETS_max_ increased significantly with 0.1, 0.5, and 1 mM butyrate compared with no butyrate (Figure 1B). Basal respiration also increased in LPS‐exposed LBs after treatment with butyrate, although these increases were not statistically significant. After treatment with each concentration of butyrate in the 24‐h rest period, mitochondrial respiration was not different between LBs incubated in control media or LPS. CS activity did not change with addition of butyrate in LBs incubated in control media or LPS (Figure 2B).

## 4 DISCUSSION

We found that sodium butyrate both prevented and reversed the LPS‐induced reduction in mitochondrial respiration in LB cell lines. Specifically, butyrate increased oxygen consumption related to ATP production, including ATP‐linked respiration and maximal respiratory capacity (ETSmax) in LPS‐exposed LBs, while this effect was less apparent in LBs exposed only to control media. The increase in mitochondrial respiration was not dependent on an increase in mitochondrial content measured by CS activity.

Our findings are consistent with prior studies demonstrating enhanced mitochondrial function after butyrate. For example, Rose et al.^12^ observed increased mitochondrial respiration with butyrate in LB cells lines from children with autism exhibiting baseline mitochondrial abnormalities. Gao et al.^4^ demonstrated increased mitochondrial biogenesis and fatty acid oxidation in mice after butyrate supplementation. Donohoe et al.^3^ was able to rescue mitochondrial oxidative phosphorylation and reset ATP levels in colonocytes from germ‐free mice after colonization with a butyrate‐producing bacterial strain. These studies support that butyrate augments mitochondrial function in several ways, including upregulation of mitochondrial biogenesis, stimulation of intermediary metabolism, reduced autophagy, and direct use of butyrate as a mitochondrial fuel. It is also possible that butyrate may modulate mitochondrial function via epigenetic modifications through its role as a histone deacetylase (HDAC) inhibitor.^6^, ^13^

Prior studies that link butyrate with various aspects of immune health suggest that butyrate is an effective immunologic modifier.^2^, ^7^, ^14^ For this report, we utilized LB cell lines rather than donor PBMCs in order to ensure a continuous source of identical cells cultured under consistent conditions. While mitochondrial respiration is three times higher in LBs compared with PBMC from children with sepsis, the relative 20%–30% decrease in respiration evident 24 h after LPS exposure (compared with control media) is similar to the differences in PBMC from children with versus without sepsis.^9^ Despite this similarity, it is not yet clear whether butyrate would have a similar effect on mitochondrial function in a broader selection of immune cells.

Our findings, while preliminary, support a mitochondrial‐based mechanism through which butyrate could augment immune health and potentially modulate organ dysfunction in sepsis. In addition to exogenous butyrate supplementation, attention to normalizing the microbiome and provision of complex carbohydrates during illness may help to optimize endogenous butyrate production. Further studies are warranted to test the role for butyrate to alter the clinical course of sepsis through its effect on mitochondrial dysfunction, both in response to and independent from its role as an HDAC inhibitor. Although in this cell model improved mitochondrial respiration did not appear to require mitochondrial biogenesis, studies using additional measures of mitochondrial content are necessary to confirm our preliminary findings using CS activity.

## CONFLICT OF INTEREST

The authors have declared that no conflicts of interest exist.

## AUTHOR CONTRIBUTIONS

**Scott L. Weiss:** Conceptualization (equal); Data curation (equal); Formal analysis (lead); Funding acquisition (equal); Investigation (equal); Methodology (equal); Writing – original draft (lead); Writing – review & editing (equal). **Donglan Zhang:** Data curation (equal); Methodology (equal); Writing – review & editing (equal). **Sumera Farooqi:** Data curation (equal); Project administration (lead); Writing – review & editing (equal). **Douglas C. Wallace:** Conceptualization (equal); Investigation (equal); Methodology (equal); Supervision (equal); Writing – review & editing (equal).

## Data Availability

The data that support the findings of this study are available from the corresponding author upon reasonable request.
